# X-ray based virtual histology allows guided sectioning of heavy ion stained murine lungs for histological analysis

**DOI:** 10.1038/s41598-018-26086-0

**Published:** 2018-05-16

**Authors:** Jonas Albers, M. Andrea Markus, Frauke Alves, Christian Dullin

**Affiliations:** 1 Institute for Diagnostic and Interventional Radiology, University Medical Center Göttingen, Göttingen, Germany; 2 Translational Molecular Imaging, Max-Planck-Institute for Experimental Medicine, Göttingen, Germany; 3 Clinic for Haematology and Medical Oncology, University Medical Center Göttingen, Göttingen, Germany; 4 Elettra Sincrotrone Trieste, Trieste, Italy

## Abstract

Examination of histological or immunohistochemically stained 2D sections of embedded tissue is one of the most frequently used tools in biomedical research and clinical routine. Since to date, targeted sectioning of specific regions of interest (ROI) in the sample is not possible, we aimed at developing a guided sectioning approach based on x-ray 3D virtual histology for heavy ion stained murine lung samples. For this purpose, we increased the contrast to noise ratio of a standard benchtop microCT by 5–10-fold using free-propagation phase contrast imaging and thus substantially improved image quality. We then show that microCT 3D datasets deliver more precise anatomical information and quantification of the sample than traditional histological sections, which display deformations of the tissue. To quantify these deformations caused by sectioning we developed the “Displacement Index (DI)”, which combines block-matching with the calculation of the local mutual information. We show that the DI substantially decreases when a femtosecond laser microtome is used for sections as opposed to a traditional microtome. In conclusion, our microCT based virtual histology approach can be used as a supplement and a guidance tool for traditional histology, providing 3D measurement capabilities and offering the ability to perform sectioning directly at an ROI.

## Introduction

X-ray based virtual histology is an emerging technique that can provide high resolution 3D data sets of small tissue samples. If combined with *ex-vivo* heavy ion staining protocols, even soft-tissue specimens can be imaged in great detail, as already demonstrated in various applications^1–3^. One of the most commonly applied staining protocols for this purpose is based on phosphotungstic acid (PTA). We previously demonstrated that PTA staining performed on paraffin embedded soft tissue samples does not interfere with subsequent histology and immunohistochemistry (IHC)^1^. A main disadvantage of virtual histology is that to date no staining procedures exist for CT imaging that allows a specific targeting of certain soft tissue structures. In contrast, traditional histology and IHC provide a multitude of staining protocols for the specific identification of certain structures or cells within the tissue. However, these can in most cases only deliver 2D images. Furthermore, histological sectioning is restricted by the limited visibility of the sample within the embedding material which prevents the localisation or identification of the region of interest (ROI). Thus, obtaining a histological analysis of a specific ROI usually requires serial sectioning and staining, which can be labour intensive and may lead to non-optimal results. For sectioning of both, hard and soft tissue samples, femtosecond laser microtomes can be applied, which can provide histological sections without any deformation of the sample^4^. In this case, the sample is cut by a laser beam rather than a knife, which does not put any mechanical stress on the sample.

In order to reach a satisfactory resolution for x-ray based virtual histology with a sufficient image quality, a high amount of x-ray photons is needed. This requirement is given for synchrotron radiation or for costly x-ray microscopes^5,6^. However, the former is often unavailable to researchers or clinicians, while the latter requires long scanning times. It has been demonstrated that instead of using pure attenuation based contrast, exploiting the phase properties of the x-rays leads to a higher image quality especially in weakly absorbing samples^7^. Several phase-sensitive techniques have been reported. Among these, free propagation phase contrast (propagation based imaging, PBI) is by far the simplest method as it does not require any additional optical elements but only a sufficiently large sample-to-detector distance^8,9^. If partially coherent x-rays are provided, as in the case of synchrotron light sources and micro-focus x-ray tubes, a local self-interference can be measured, which is generated by the refraction of the x-ray beam at tissue interfaces. This interference creates edge enhancement of the projected tissue structures if recorded in the near-field Fresnel regime. In combination with a single distance phase retrieval algorithm the real part of the local refractive index within the sample that describes the phase shift can be calculated from the recorded edge enhanced projection data sets. However, the algorithm can only deal with objects that demonstrate an a-priori known constant ratio between attenuation and refraction, meaning the real and imaginary part of the complex refractive index^10^. This requirement is certainly not fulfilled in biological samples, however it has been demonstrated that results are stable if the range of the atomic numbers differs by a maximum of 11, as is the case in soft tissue^11^. Therefore, this approach became the standard protocol for biomedical research at many synchrotron tomographic beamlines. Because partial coherence of the x-rays is required, clinical CT systems cannot be used. So far only synchrotrons and x-ray microscopes meet this requirement^6^. Here we aimed to evaluate the applicability of PBI in a standard microCT system using geometric magnification.

In order to exploit the full potential of a combination of 3D virtual and 2D classical histological analysis a precise match of both data sets is needed. However, such an approach is complicated by the non-uniform deformations of the histological slices that can occur during mechanical sectioning. Moreover, the very different image content of specifically stained histological sections, for instance PTA stained CT volume data sets, add to this challenge. Therefore our second objective was to develop a novel approach that combines block matching^12^ between the virtual section and the microscope image to quantify local displacement with the calculation of the mutual information^13^ between these blocks. Mutual information is defined as the reduction in uncertainty about one variable given the knowledge of another. In our case, the grey value of one image compared to the grey value in the other image at the same location. In case of a perfect match the mutual information is maximized, which means that the grey values at a certain position are homogenous in both images. The developed algorithm calculates the novel “Displacement Index” which can be used to quantify the quality of the overlay of virtual and mechanical cuts and therefore presents a measure of the reliability of the performed hybrid approach.

Here we present a virtual histology approach which uses microCT data sets of stained and embedded murine lung samples as a priori information to guide the sectioning process on the example of mouse lungs. We show that employment of a standard microCT device and exploitation of phase contrast for improvement of image quality allows the accurate determination of optimal cutting positions and thus provides all necessary prerequisites to the future development of a computer guided sectioning system.

## Results

### Integration of high resolution microCT into the traditional histological workflow

Based on our previous results, which demonstrate that PTA staining and microCT imaging of tissue specimens does not interfere with subsequent histology or IHC and can be applied to various organs including mouse heart, kidneys, brain and entire embryos^1,14–16^, the aim of this study was to further explore the possibilities of using microCT imaging to improve the workflow of histological analysis.

**Figure 1:**
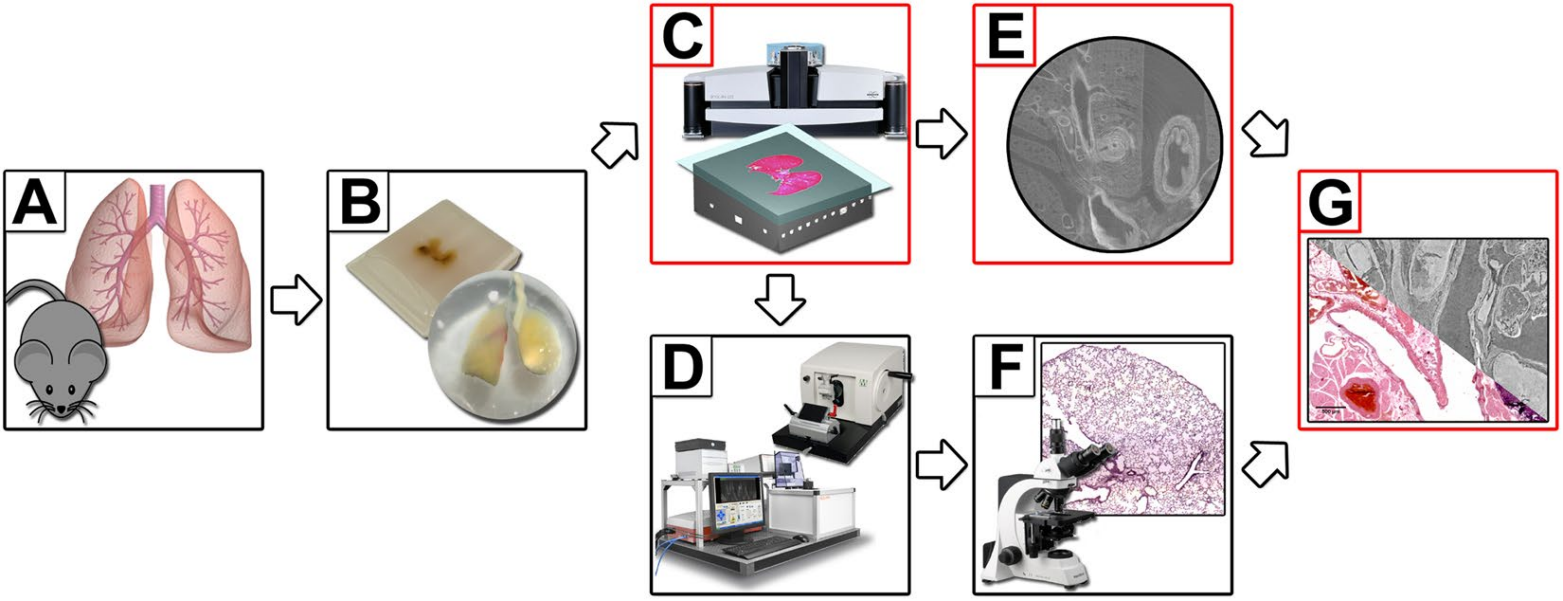
Workflow of microCT guided sectioning. (**A**) Lungs were dissected from FVB mice. (**B**) Specimens were PTA stained, dehydrated and embedded either in paraffin or resin. (**C**) A microCT scan of each sample was performed and the position of interest defined within the 3D reconstructed phase retrieved data sets. (**D**) The samples were cut at the predefined ROI using either a standard microtome (paraffin) or cutting by grinding in combination with the laser microtome (resin) (left, TissueSurgeon, LLS ROWIAK LaserLabSolutions, image use permitted by LLS ROWIAK LaserLabSolutions under the Creative Commons Attribution License CC BY 4.0). (**E**) and (**F**) A virtual slice and a microscope image of the PTA stained lung tissue slices were produced and (**G**) the “degree of matching” was analysed using the developed block matching approach.

For this purpose, we integrated high resolution microCT imaging directly into the traditional histological workflow and thus implemented a tool which allows sectioning at predetermined ROIs. Figure 1 shows the workflow with integrated microCT. Steps that were added to the standard histological workflow are shown in red boxes (Fig. 1C,E and G). Following dissection of the mice (Fig. 1A), the lungs were stained with PTA, dehydrated and embedded in either paraffin or resin (Fig. 1B). Both samples were then analysed by microCT (Fig. 1C). Furthermore, we applied PBI to improve the image quality of the obtained virtual histology data sets. The microCT data sets of the embedded lung tissue samples were then used to define the sectioning to specific location using a normal microtome for the paraffin sample and a diamond saw in combination with a laser microtome for the resin sample (Fig. 1D). The location of the section was determined by choosing an ROI in the CT data set with the help of 3D rendering software. The distance of the ROI to the surface of the embedding material in the Z-axis was measured and used to calculate a cutting scheme to precisely reach the desired ROI (Supplemental material 1). In order to quantify the quality of the approach, the matching degree between the resulting virtual cut (Fig. 1E) and stained histological tissue slices (Fig. 1F) was then calculated using an adapted block matching approach described below (Fig. 1G).

### Inline free propagation phase contrast is possible in a standard bench-top microCTs and leads to a substantial gain in image quality

**Figure 2:**
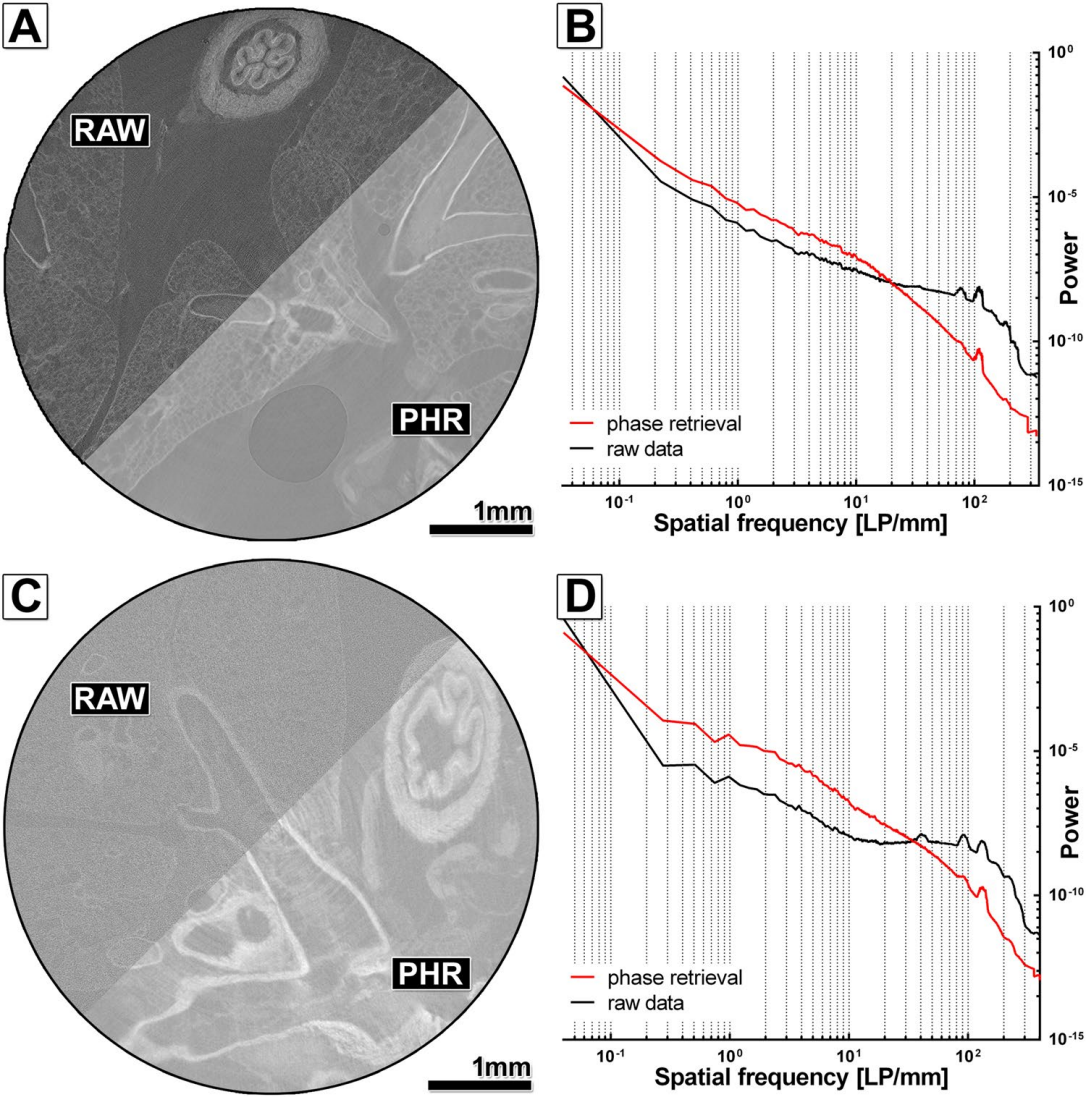
Free propagation phase contrast in high resolution microCT of phosphotungstic acid (PTA) stained paraffin or resin embedded mouse lungs. (**A**) Displays the comparison of the reconstruction based on RAW and PHR for the PTA stained and paraffin embedded mouse lung sample. An increased contrast and slightly lower noise level can be found in the PHR reconstruction. (**B**) Shows the radially averaged power spectra (raPS) for the images shown in (**A**). The PHR curve (red curve) shows higher intensities in the medium frequency range and lower intensities in the high frequency range. However, the difference is smaller than in (**D**) for the resin embedded sample. (**C**) Shows the comparison of the reconstructed raw data (RAW) and the phase retrieved (PHR) data (ANKA phase δ/β ratio 100) of a PTA stained mouse lung embedded in resin. A higher contrast and a lower noise level can be clearly seen in the phase retrieved data. (**D**) The raPS show a higher power in the medium frequency range and a lower power at the cut-off frequencies for the PHR data set (red curve) compared to the RAW data set (black curve), pointing to steeper edges and lower noise level.

To improve the image quality of the microCT scans we evaluated the possibility of using PBI in combination with a single distance phase retrieval algorithm, an established method to increase the contrast to noise ratio (CNR) in synchrotron microCTs and x-ray microscopes and, especially suited for low absorbing samples. Key factors for this method, which does not utilize any additional x-ray optical elements, are: (a) a sufficient degree of spatial coherence, (b) a sample-to-detector distance (SDD) large enough to allow the formation of self-interference patterns of the x-ray wave front distorted by the refraction with the sample and (c) a small pixel size to record the phase effects together with the absorption-based contrast. Here we used SDDs in the range of 218 to 227 mm and pixel sizes of 1.25–1.5 µm. Figure 2A shows the difference in the appearance of the reconstructed raw data set and the reconstructed phase retrieved data for the PTA stained and paraffin embedded sample. The δ/β-ratio of 100 used in the “ANKAphase” software was adjusted to remove any phase contrast related edge enhancement. Provision of a theoretical derived δ/β-ratio is complicated by two factors: (a) the inhomogeneous composition of the specimen and (b) the usage of polychromatic x-rays with an unknown spectrum. Despite these obstacles, we achieved a 5.3-fold gain in the CNR in the phase retrieved data set of the paraffin embedded lung sample (Table 1). Moreover, the power spectra in Fig. 2B demonstrate an increased power in the medium spatial frequencies and a reduction in the cut-off frequencies for phase retrieved data (red curve) compared to raw data (black curve), pointing to sharper edges and a reduced noise level in phase retrieved data sets. Figure 2C demonstrates the same visual comparison between the reconstructed data sets of the resin embedded lung sample. Here an 11.3-fold increase in the CNR could be achieved (Table 1). Figure 2D shows that phase retrieval in the resin embedded sample improves image quality even more, because a pixel size of 1.25 µm was used instead of 1.5 µm for the paraffin embedded one. This verifies that in addition to the SDD, the pixel size is crucial for the applicability of phase contrast imaging. In classical microCTs, small pixel sizes are generated by geometric magnification which requires positioning the sample close to the source. Therefore, maximum resolution can only be reached for small specimens. We used the smallest possible sample-to-source distance for both samples, which resulted in different pixel sizes and SDDs due to the different diameters of the samples. In summary, free propagation phase contrast imaging in combination with single distance phase retrieval provided a significant improvement in the image quality of both, the paraffin and the resin embedded tissue.

**Table 1:** Contrast-to-noise ratios (CNR) between tissue and embedding material for the reconstructed raw data set (RAW) and the reconstructed phase retrieved data set (PHR) for the PTA stained lungs embedded in resin and paraffin.

	Paraffin	Resin
CNR RAW	1.90 ± 0.45	0.51 ± 0.05
CNR PHR	9.98 ± 1.65	5.94 ± 1.33
CNR PHR/CNR RAW	5.3 ± 1.73	11.6 ± 3.06

The ratio (CNR PHR/CNR RAW) reveals that phase retrieval was more beneficial for the resin embedded sample than for the paraffin embedded sample, but in both cases significantly improved the CNR.

### Laser cut sections show a higher degree of matching with CT data sets than traditional histological sections

**Figure 3:**
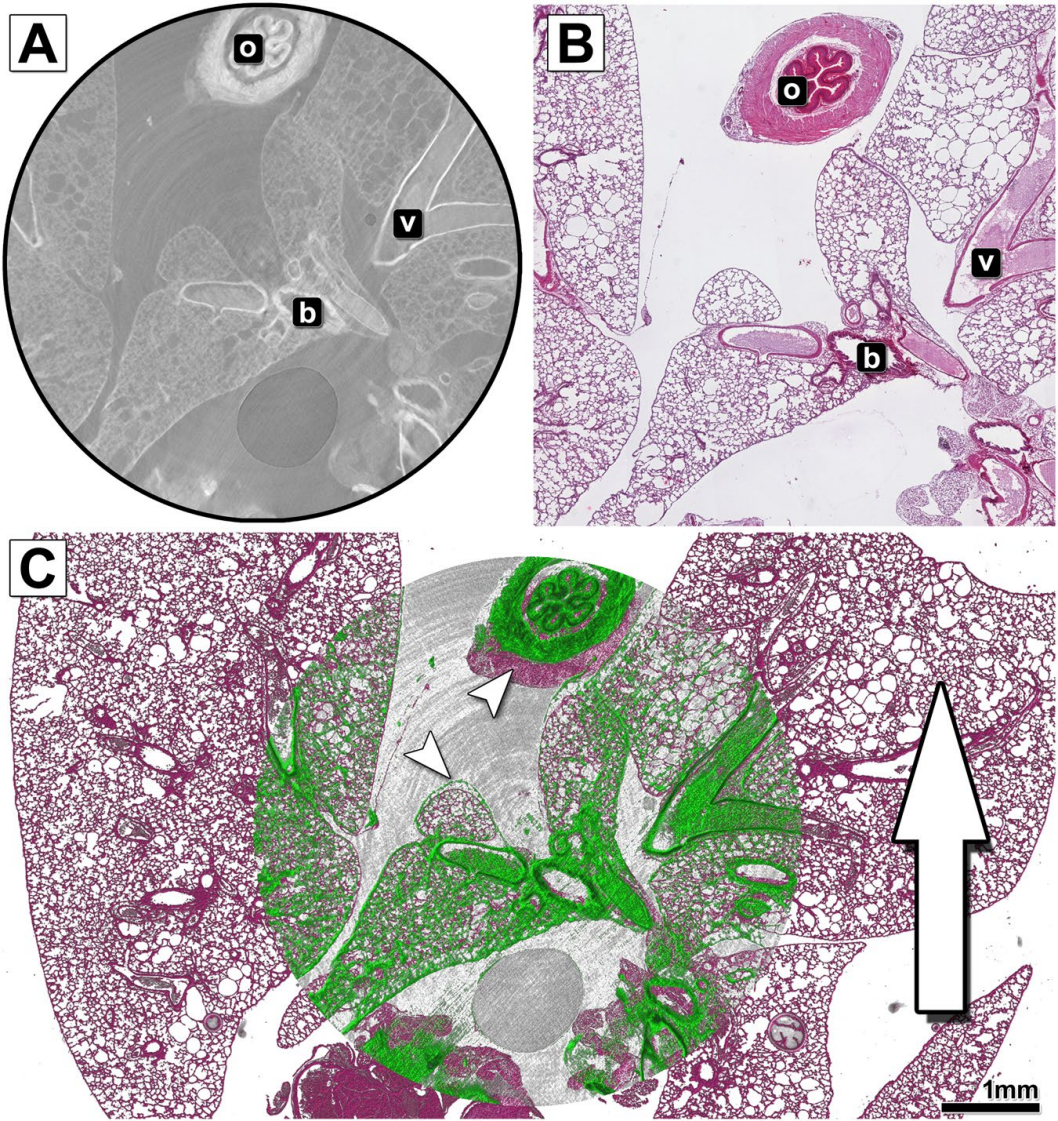
Comparison and overlay of the microCT scan and histological image of the paraffin embedded and microtome cut mouse lung sample. (**A**) Shows an exemplary slice of the reconstructed PHR microCT data set of the paraffin embedded PTA stained mouse lung. In addition to alveolar lung tissue, the oesophagus (o), larger bronchi (b) and blood vessels (v) are displayed. (**B**) Shows the microscope image of the haematoxylin & eosin (HE) stained tissue section cut with a classic microtome at the predefined position. (**C**) Depicts the overlay of both data sets. The histological data is shown in pseudo HE colours (the data set was colourised for better visualisation), while the CT data is shown in green. The white arrow represents the cutting direction of the microtome. The strongest deformation (indicated by white arrowheads) clearly appears in that axis, especially at the site of the oesophagus. The quality of the overlay is strongly reduced when compared to the laser cut specimen.

In order to analyse the degree of matching between traditional and virtual histology we overlaid the virtual section from the CT data set of the PTA stained paraffin embedded mouse lung (Fig. 3A) with the haematoxylin & eosin (HE) stained histological slice performed at the same position (Fig. 3B). The result is displayed in Fig. 3C and reveals that the histology (red) and CT image (green) did not match precisely and showed non-uniform deformations in the HE stained slice (white arrowheads). When considering the cutting direction of the microtome (white arrow) it becomes apparent that the strongest deformations and local compression occurred in the direction of the cutting process. Especially the morphology of the oesophagus, which is not connected to other tissues within this slice, experienced a major displacement. More rigid structures such as larger bronchi or vessels, did not show major displacements from the alveolar lung tissue.

**Figure 4:**
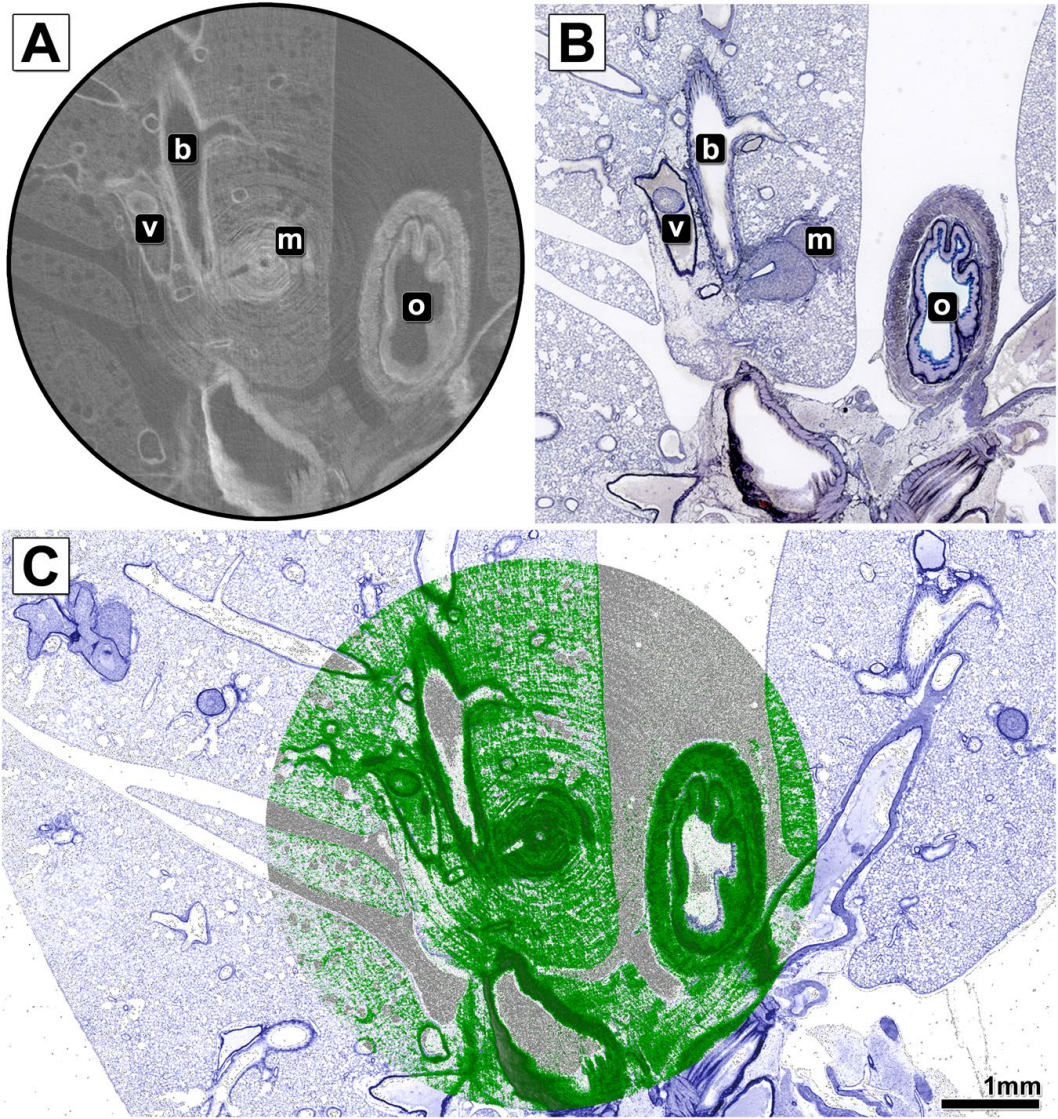
Comparison and overlay of the microCT scan and histological image of the resin embedded and laser cut mouse lung sample. (**A**) Shows an exemplary slice of the reconstructed PHR microCT data set of the resin embedded PTA stained mouse lung. In addition to alveolar lung tissue, a lung metastasis (m), the oesophagus (o), larger bronchi (b) and blood vessels (v) are displayed. (**B**) Shows the microscope image of the Sandersons Rapid Stain (SRS) stained and van Gieson (VG) counterstained cut performed with the laser microtome at the predefined position. No cutting artefacts are visible. (**C**) Depicts the overlay of both data sets. The histological data is shown in pseudo SRS/VG colours (the data set was colourised for better visualisation), while the CT data is shown in green. The overlay of the CT data with the histology image reveals a precise match of both data sets.

By contrast, the overlay of the virtual histology data of the resin embedded specimen (Fig. 4A) with the corresponding Sandersons Rapid Stain (SRS) stained and van Gieson (VG) counterstained laser cut slice (Fig. 4B) shown in Fig. 4C (CT image green, histology image blue) demonstrates a nearly perfect match between the data of both modalities.

An excellent example for the application of CT guided virtual histology is demonstrated in the same Figure: The lung metastasis, which was the desired ROI, was sectioned straight through its centre, which had not been possible by classical serial sectioning. Moreover, our microCT approach supplements the histological analysis by 3D parameters and thus allows the calculation of the volume of the tumour lesion (for this particular metastasis, a volume of 0.47 mm³). The volume of the metastasis was measured using a threshold based segmentation in a 3D ROI around the metastasis.

### Quantification of the matching degree between CT and histological data sets using the “Displacement Index” (DI)

**Figure 5:**
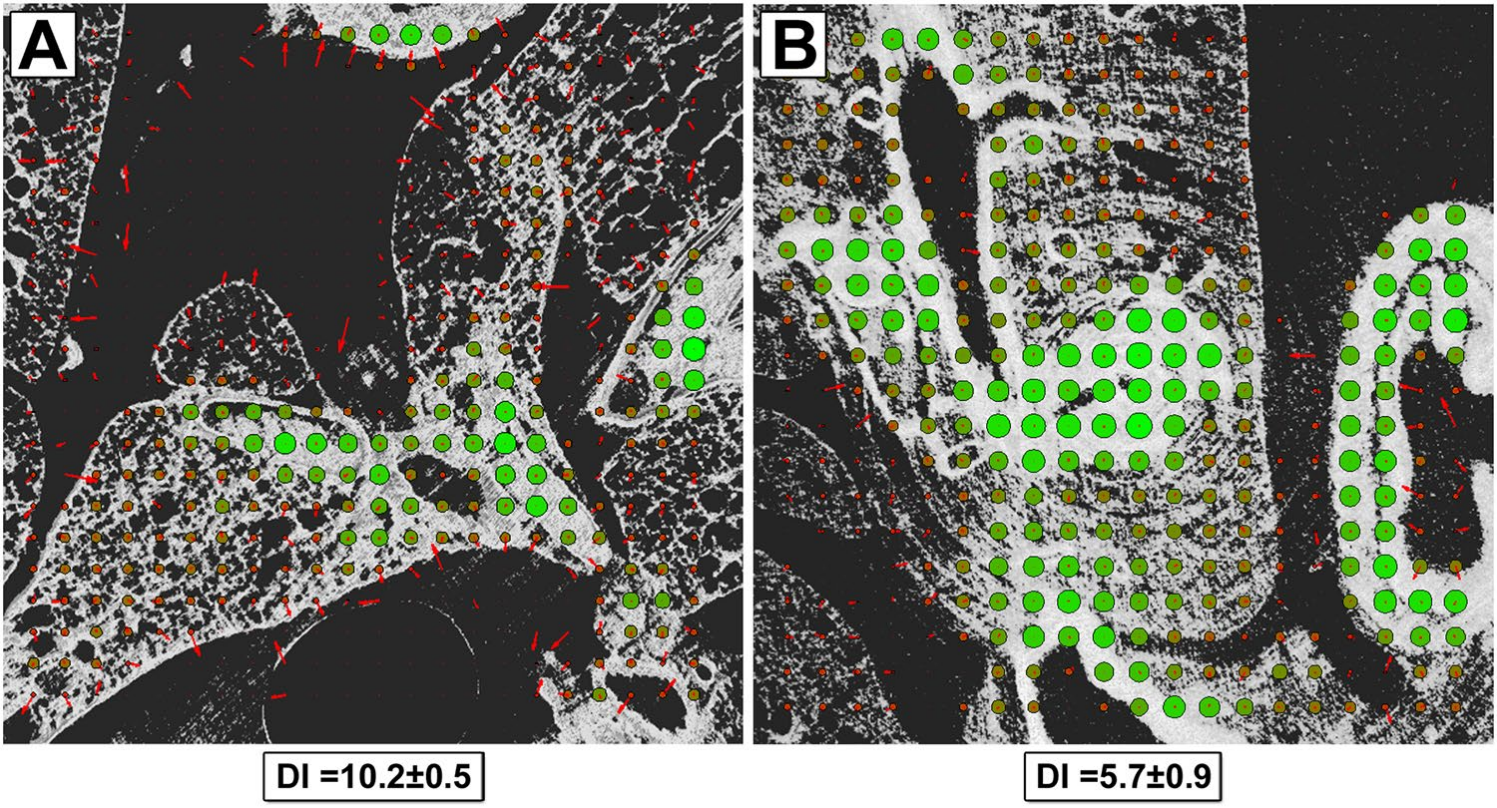
Quantitative analysis of the matching quality between histological data and microCT data for both, the laser and the microtome cut tissue sections: (**A**) and (**B**) show the results of the block matching analysis between the PHR CT data of (**A**) the paraffin embedded and (**B**) the resin embedded mouse lung with their corresponding histological images. The local displacement vectors are depicted in red, while the magnitude of mutual information (MI) is illustrated in size and in colour (from red to green) of the circles. A higher value of MI indicates a closer match between the structures in CT and histology. Areas with less than 1% of brightness were not processed. In (**B**), the resin embedded tissue, nearly no local displacement (despite some areas dominated by noise from the embedding material in the CT scan) and a higher degree of local MI are found compared to (**A**) the paraffin embedded lung. The overall displacement index DI is a lot lower for the laser cut resin sample (DI = 5.7 ± 0.9; n = 4) than for the paraffin sample cut with the standard microtome (DI = 10.2 ± 0.5, n = 4).

For further quantification of the matching precision between the histological analysis and the corresponding virtual cut in the CT data set, we were faced with two problems: a) the encountered deformations in the classic histological paraffin slice are non-uniform and b) the image content in both modalities – classic histology and CT – is related to the used staining procedure (HE and PTA) and does therefore not necessarily correspond to each other. To deal with these problems we developed a novel analysis scheme (described in more detail in Supplemental material 3). To verify the presence of non-uniform deformations we performed a block matching that analyses the local displacement and degree of matching for small blocks of 50 × 50 points each. To facilitate the comparison of both types of images, data sets were downscaled to the same spatial resolution and transferred to a grey scale range of [0, 1]. For calculating the local displacement, we used the cross-correlation between two corresponding tiles implemented in the Fourier domain. To avoid that spatial frequencies describing the size of the artificially sectioned blocks dominate the Fourier space, the tiles were multiplied with a circular kernel function that generated smooth edges (Supplemental material 3). Moreover, the tiles were embedded in a matrix twice their size in each dimension to avoid edge effects in the correlation function. After the local displacement was identified, the tile of the histological data was translated accordingly and then the local mutual information (MI)^8^ was calculated. Based on the joint entropy, the MI becomes maximal if homogeneous structures in both modalities match each other independently of their respective grey value. The results are displayed in Fig. 5 for (A) the paraffin embedded tissue slice with a standard microtome and (B) the resin embedded and laser cut sample. The local displacement is shown with red arrows, the magnitude of the local MI by the size and colour (green = high, red = low) of the displayed circles. In the case of blocks that contained less than 1% structure, no calculation was performed (displayed as small red dots). Example DI calculations to validate the method can be found in Supplemental material 4. Clearly, less local displacement and higher MI are visible in the resin embedded sample. The minor non-zero length displacement vectors are mostly related to background noise in the CT data set created by impurities in the embedding material. The paraffin sample showed larger local displacements, indicated by the red arrows. Moreover, the arrows are not oriented in a single direction, indicating non-uniform deformations, which cannot be explained with a misalignment of the data sets. For quantitative comparison, we defined the “Displacement Index” (DI) that averages all local displacements weighted by the inverse MI (measured in bits), as we believe that a displacement which leads to a high degree of match (high MI) should be penalised less. Thus, the DI becomes minimal for more precise fitting data sets. We found that the DI of the laser cut sample was much smaller (DI = 5.7 ± 0.9; average ± standard deviation of 4 analysed pairs of virtual and histological sections) than the one sectioned by the standard microtome (DI = 10.2 ± 0.5; average ± standard deviation of 4 analysed pairs of virtual and histological sections). This difference in the generated DIs underlines that the laser cut sample displays a much larger correspondence to the virtual slice of the x-ray based virtual histology data set than the paraffin sample cut with a classic microtome.

## Discussion

Here we present a virtual histology approach based on microCT acquisitions of phosphotungstic acid (PTA) stained and embedded murine lung samples. We demonstrate that this method integrates well in the standard workflow of classical histology and provides a detailed 3D representation of the tissue sample. These 3D data sets can be used to precisely determine the cutting position in an ROI, thereby eliminating labour-intensive serial sectioning. Moreover, we show that traditional microtomes introduce substantial non-uniform deformations in the tissue slices, an effect that can be avoided by utilizing a laser-microtome.

We demonstrate that PBI^9,17,18^ can be successfully used in state-of-the-art bench-top microCTs. PBI is known to generate data sets with an increased CNR compared to classical attenuation based CT, especially for weakly absorbing objects^7^. Moreover, PBI does not require any additional optical elements like other phase sensitive techniques such as grating based interferometry. This not only makes PBI more dose efficient but also less costly in its implementation. The main difference between synchrotron based microCT and classical microCT is that in the latter case the high resolution is generated by geometric magnification, i.e. using an x-ray beam with a high cone angle instead of a parallel beam. Recent developments such as liquid metal jet x-ray tubes^19^ or x-ray microscopes^6^ have enabled the application of PBI under non-synchrotron conditions. Bidola *et al*.^6^, claim to use free propagation phase contrast in classical microCT. However, only results are demonstrated using an x-ray microscope setup with Fresnel lenses. This allows longer sample-to-detector distances due to a much smaller cone angle. Thus, we believe our approach is the first to utilize PBI in a commercially available standard microCT.

Gradl *et al*.^20^ state that the SDD of a classical cone beam microCT has to be at least four times longer than that of a synchrotron setup (or compact light source) for the same experiment. Nevertheless, we were able to successfully apply PBI in a cone-beam microCT with a limited SDD based on the fact that the effective detector pixel size, which is crucial for the detection of the phase effects, scales in the same way as the SDD due to geometric magnification. We demonstrate that using SDDs in the range of 20 cm can be sufficient to observe phase effects in a standard bench-top microCT. By exploiting these effects, we achieved a 5 to 11-fold gain in CNR. We do however suggest that future microCT systems incorporate the possibility for larger SDDs by enlarging their cabinets. PBI could then play a major role in providing higher quality microCT images at high resolution.

Our virtual histology approach was enabled by the *ex-vivo* PTA staining of soft-tissue specimens, a process that greatly enhances the x-ray absorption of the sample and generates contrast in dependence of a combination of specific binding to collagen, fibrin and extracellular matrix as well as differences in the diffusion process through adjacent structures. A number of successful CT based analyses of PTA stained soft-tissue has been reported^19–21^. However, this approach has two major limitations: a) the slow diffusion speed of the heavy tungsten ions results in long staining periods in the range of several days, even for small specimens like mouse organs^1^ and b) the edge definition of tissue interfaces is lowered by the PTA diffusion. Therefore, this approach is limited to applications in which sample processing time is not a pressing issue. However, x-ray based virtual histology enables the guidance of the cutting process to ROIs, which renders serial cutting unnecessary and in addition ensures that important structures are not missed. In our opinion, this will compensate for the additional time that is needed for the PTA staining process and leads to a dramatic reduction of the workload in histology laboratories. Our technique is not yet automated, but since precise geometric locations of ROIs can be derived from the CT data sets of the scanned embedded samples, it is possible to develop computer controlled cutting systems that mechanically achieve the virtually planned sectioning. Such systems could be either based on a microtome or a diamond saw depending on whether soft tissue or hard tissue needs to be processed.

Another important difference between CT based virtual and standard histology is the fact that to date no specific radio-opaque *ex-vivo* staining procedures exist. It has been demonstrated by Descamps *et al*.^22^ that different protocols (Osmium, PTA, Phosphomolybdic acid (PMA)) lead to differently contrasted structures, however none of these methods is as specific as for instance IHC. Since some of the x-ray staining protocols are based on different heavy elements it might be worthwhile combining them and use spectral CT^23^ to assess the specific separation of structures. In addition, x-ray based virtual histology can be used to more easily derive geometric and volumetric anatomical measures as compared to classic histology, as shown in our example of the volumetric analysis of a lung metastasis. More importantly, we demonstrate that standard microtomes introduce major non-uniform deformations within the tissue slices, which in our opinion makes studies based on CT measures much more reliable.

In this work the feasibility of the x-ray based virtual histology approach was demonstrated using murine lungs. It has been shown that lung tissue experiences one of the strongest deformations among tissue types during the mechanical sectioning^24^. Thus we believe lung tissue is ideal to demonstrate the benefits of virtual histology and sectioning with a laser microtome. Because the preparation of biological samples for histological analysis is very similar for most tissues, we believe that the proposed guided sectioning approach is generally applicable. It has to be noted, that staining times for larger samples and samples with higher density can be longer than for the used murine lung samples.

In conclusion, we established a novel approach of using free propagation phase contrast microCT based virtual histology of *ex-vivo* stained and paraffin or resin embedded murine lung samples to determine the cutting position for histological analysis. The demonstrated precise fit of laser-cut tissue slices with the microCT data validates that both modalities, virtual and classic histology, complement each other with their respective unique information. This procedure not only enables the improvement of histological analysis, but also offers the potential for automation and thus a dramatic reduction of the workload in clinical routine.

## Methods

### Staining and embedding

Lungs of two female FVB/N-Tg (MMTVneu)202Mul/J mice, a strain which can spontaneously develop lung metastasis, were explanted, briefly rinsed in water and stained with phosphotungstic acid (PTA) for one day, according to the protocol described by Dullin *et al*.^1^. One sample was embedded in paraffin (standard histology paraffin block of about 2.5 × 3.5 × 1 cm^3^) and the second sample was embedded in Methyl-Methacrylate (MMA) following chemical dehydration.

### MicroCT acquisition and phase retrieval

MicroCT data sets were acquired using a SkyScan 1272 microCT (Bruker MicroCT). The paraffin embedded lung sample was scanned using a tube voltage of 90 kV, a tube current of 110 µA, 1800 projections in a 180° scan, an SDD of 227 mm and a 1.25 µm pixel size. The resin embedded lung sample was scanned using a tube voltage of 90 kV, a tube current of 110 µA, 3600 projections in a 180° scan, an SDD of 218 mm and a 1.5 µm pixel size.

In order to retrieve the phase information from the recorded projection data we used single distance phase retrieval based on the Paganin algorithm^8^ implemented in the ANKAphase ImageJ plugin with the following parameters: δ/β ratio of 100, SDD of 218 to 227 mm, energy of 45 keV and pixel size of 1.25 to 1.5 µm.

### Quantitative assessment of image quality

For assessment of image quality, the ratio between the contrast of two adjacent structures and the noise level are major criteria, as it resembles the ability to visualize different structures. Here we define contrast-to-noise ratio (CNR) between two tissues (1 and 2) according to the definition of van Engen *et al*.^25^ as $CNR=|{S}_{1}-{S}_{2}|/\sqrt{0.5({\sigma }_{1}^{2}+{\sigma }_{2}^{2})}$ with S the average grey value in a homogeneous region and $\sigma$ the standard deviation in the same region. As the CT system utilizes a cone-beam geometry, slices more distant to the optical axis show a different contrast-to-noise ratio than those in the centre. To account for this, we measured the CNR of 3 slices distributed equally throughout the entire data set. A region in the embedding material and three regions within the muscle tissue of the oesophagus were measured in each slice. The oesophagus was chosen, because it provides a near homogeneous structure large enough for a reliable measurement. The CNR for each slice was calculated separately. Table 1 shows the mean CNR value of the different slices and their standard deviations.

Since CNR depends on the noise level - which can easily be lowered by low pass filtering - it is important to simultaneously judge the image sharpness. For this purpose, we used power spectra analysis. A theoretical explanation of the used power spectra is given in Supplemental material 2.

### Sectioning techniques

The PTA stained and paraffin embedded mouse lung was sectioned in slices of 2 µm thickness at the predefined position in the microCT data set using a HM 340 E microtome (Thermo Fischer Scientific). Sections were mounted on glass slides.

The resin embedded lung sample was first cut at the predefined position based on the analysis of the CT data using an Isomet 1000 Precision diamond saw (Buehler). Subsequent polishing with silicone carbide paper provided a coplanar even surface. This polished surface of the sample was mounted on a microscope glass slide using optical transparent cyan acrylate based glue (LLS ROWIAK LaserLabSolutions). Using the TissueSurgeon laser microtome (LLS ROWIAK LaserLabSolutions), a femtosecond NIR laser was focused 10 µm above the sample surface within the tissue, resulting in sections with a thickness of approximately 10 µm. The system generates plasma within an approximately 0.5 µm sized focal point that is moved in a raster scheme through the entire sample. Due to the ultrashort laser pulses, the minimum absorption and the low energy, no heat is generated within the sample and no mechanical force is applied, which results in a non-deformed slice.

### Histological analysis of tissue slices

Paraffin embedded lung sections were stained with haematoxylin & eosin (HE) as described before^19^. Resin embedded lung sections were stained with Sanderson’s Rapid Stain (SRS), followed by a van Gieson (VG) counterstain (Dorn & Hart Microedge, Inc). This staining procedure did not require the removal of the resin, which could otherwise generate deformations of the tissue slice. Histological images were acquired using an Axiovert 200 inverted microscope (Zeiss). To depict the whole paraffin section, the built-in stitching algorithm of the microscope was used with a 10 × magnification.

### Assessment of “degree-of-matching” between microCT and histology

In order to judge the quality of the matching between microCT based virtual histology and microscope image of the stained slices, we developed an algorithm that performs block matching based local correlation and measures the local mutual information.

The stitched microscope images (n = 4 for paraffin embedded sample and resin embedded sample) and the closest matching slices through the microCT data sets (n = 4 for paraffin embedded sample and resin embedded sample) were downscaled to the spatial resolution of the corresponding µCT images and linearly transferred to a grey scale between 0 and 1 in 8-byte floating point precision according to their individual minimum and maximum values, with 0 = black (background) and 1 = white (highest intensity). Blocks of 50 × 50 pixel size were generated and multiplied with a 2D circular kernel function to ensure circular shape and smooth edges to suppress spatial frequencies related to the block size rather than the image content. To detect the local displacement vector, a cross-correlation implemented in Fourier space was calculated. The histology image was translated according to the detected displacement vector and the local mutual information MI was calculated. MI = E1 + E2 − JE with E1 entropy of block in image1, E2 entropy of block in translated image2 and JE joint entropy of image1 and translated image2. In order to weight the calculated displacement with the mutual information, the displacement index DI was calculated. $DI=\frac{1}{G}\sum _{i}\overline{{d}_{i}}M{I}_{i}^{-1}$ with $G=\sum _{i}M{I}_{i}^{-1}\,$and $\overline{{d}_{i}}$ the magnitude of the displacement vector and $M{I}_{i}$ the mutual information of block i. The analysis was performed in a raster scheme with 50% overlapping blocks. Blocks that contained insufficient image content (for instance empty embedding material) as judged by a relative sum of grey values below 1%, were not processed. See supplemental material 2 for a more detailed description of the analysis scheme.

### Applied software

ImageJ with the ANKAphase 2.1 Plugin^26^ was used to perform phase retrieval on microCT projection data. NRecon (Bruker MicroCT, Version 1.7.1.0) was used for 3D reconstruction of raw and phase retrieved projections. The software Scry v6.0 (Kuchel & Sautter GbR) developed by the authors was used for 3D rendering of the CT data sets and for generating the virtual sections. DataViewer (Bruker MicroCT, Version 1.5.2.4) was used for the reorientation of CT datasets. CNR was measured in ImageJ. The radially averaged power spectra were generated in MATLAB (Version R2017a) using a code by Evan Ruzanski^27^. The local displacement was measured in MATLAB (Version R2017a).

### Use of experimental animals

No animal *in vivo* procedures were performed. Organs were retrieved from mice that were already sacrificed for maintenance of the institute’s breeding colony, in compliance with the guidelines of the European Directive (2010/63/EU) and the German ethical laws which were approved by the administration of Lower Saxony, Germany (33.14-42502-04-10/0017).

### Data availability

All data sets included in this work are available under: doi:10.17605/OSF.IO/3CWKY.

## Electronic supplementary material

Supplemental material
