# Supplementary material for: X-ray based virtual histology allows guided sectioning of heavy ion stained murine lungs for histological analysis
Source: Sci Rep. 2018 May 16;8:7712. doi: 10.1038/s41598-018-26086-0 (PMC5955938; doi:10.1038/s41598-018-26086-0)
Supplement: Supplementary file 1 — Supplemental material [file 41598_2018_26086_MOESM1_ESM.pdf]

X-ray based virtual histology allows guided sectioning of heavy ion stained murine lungs for histological analysis

Jonas Albers[1,*], M. Andrea Markus[2], Frauke Alves[1,2,3#], Christian Dullin[1,4#]

[1] Institute for Diagnostic and Interventional Radiology, University Medical Center Göttingen, Germany
[2] Translational Molecular Imaging, Max-Planck-Institute for Experimental Medicine, Göttingen, Germany
[3] Clinic for Haematology and Medical Oncology, University Medical Center Göttingen, Germany
[4] SYRMEP beamline, Italian synchrotron light source "Elettra", Trieste, Italy

# equal contribution

* correspondence to: jonas.albers@med.uni-goettingen.de

Supplement:

Supplemental material 1:

Explanation of the guided sectioning approach

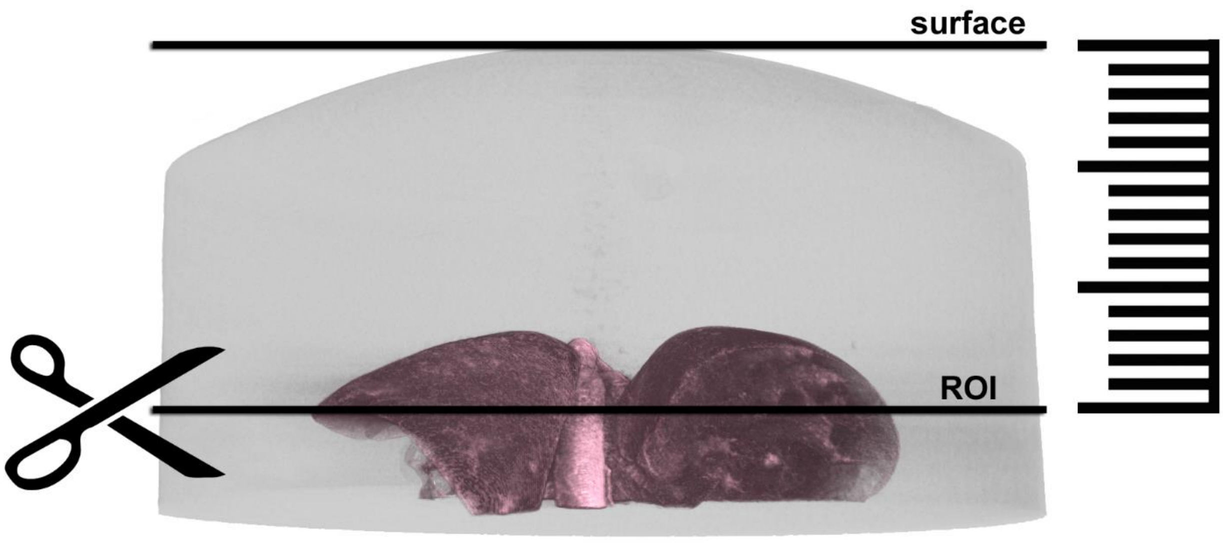

A 3D data reconstruction of the resin embedded sample is shown (scanning parameters: tube voltage 90 kV, tube current 110 µA, 1800 projections and pixel size 5.4 µm. First a region of interest (ROI) was determined in the 3D dataset. The distance from the surface of the embedding material to the ROI was measured using render software. The embedding material was cut at the measured distance with a diamond saw (resin embedded sample) or with a microtome (paraffin embedded sample).

Supplemental material 2:

Explanation of radially averaged power spectra

The content of an image can be understood as a summation of waves in space. To this end, the 2D Fourier transformation calculates the values of the corresponding frequencies. The power spectrum is the absolute value of the 2D Fourier transform normalized by the size of the analysed image and therefore represents the intensity of a certain frequency within the image. Images with steeper edges will contain higher intensities in the frequency band that describes the edge. Images with sharper edges will contain higher frequencies. Random noise can be understood as high frequency variation within the image. Thus, the intensity in the cut off region of the spatial frequencies is related to the noise level of the image. Overall, images with a higher sharpness (meaning steeper and sharper edges) and a lower noise level should demonstrate in comparison with images of lower quality, higher power in the medium to high frequency band and lower power at the cut-off frequency. In order to analyse the data independently of the orientation of the edges within the images, we present the radially averaged power spectra.

Supplemental material 3:

Explanation of DI calculation

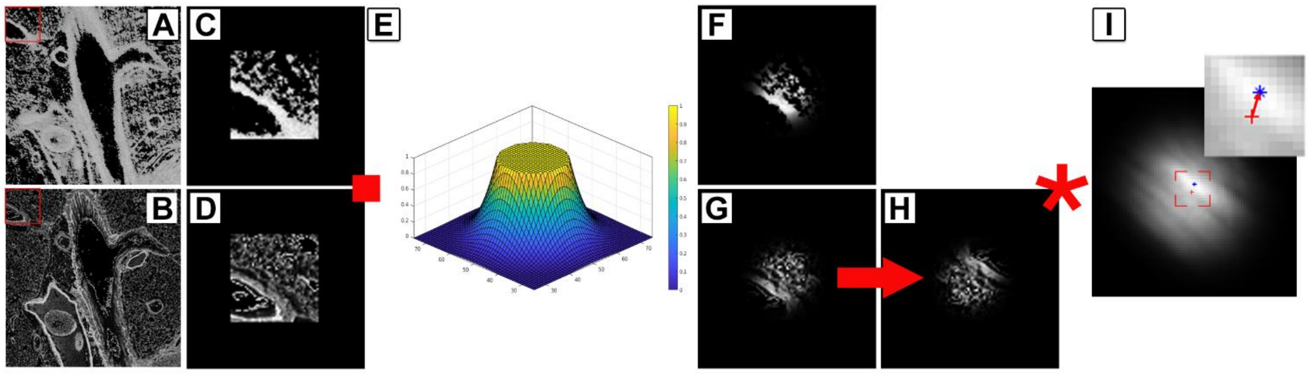

In order to analyse the degree of matching between the virtual slice through the CT data sets (A) and the microscopy image (B) we first transferred both images into the same grey scale [0, 1]. Then we analysed the images in blocks of 50x50 pixel overlapping by 25 pixels (red rectangle (A) and (B). These blocks were embedded in 100x100 pixel black images and multiplied with a 2D circular kernel function (E) resulting in the modified tiles shown in (F) and (G) respectively. Since the cross-correlation can be modelled as convolution with the mirrored and complex conjugated function, we mirrored the block (G) resulting in (H). The convolution was realized by multiplying the Fourier transformation of (F) and (H). The correlation displayed in (I) shows a maximum (blue asterisk) distant from the centre (red cross). This off-centre position represents the detected displacement between (C) and (D) and is shown as red arrow in the insert in (I). Finally, the block (G) was translated according to the detected displacement (G') and the mutual information MI = E(F)+E(G')-JE(F, G'), with E(F) the entropy of (F) and E(G') the entropy of the translated (G) and JE(F,G') the joint entropy of (F) and the translated (G) was calculated.

Supplemental material 4:

Validation of DI calculation

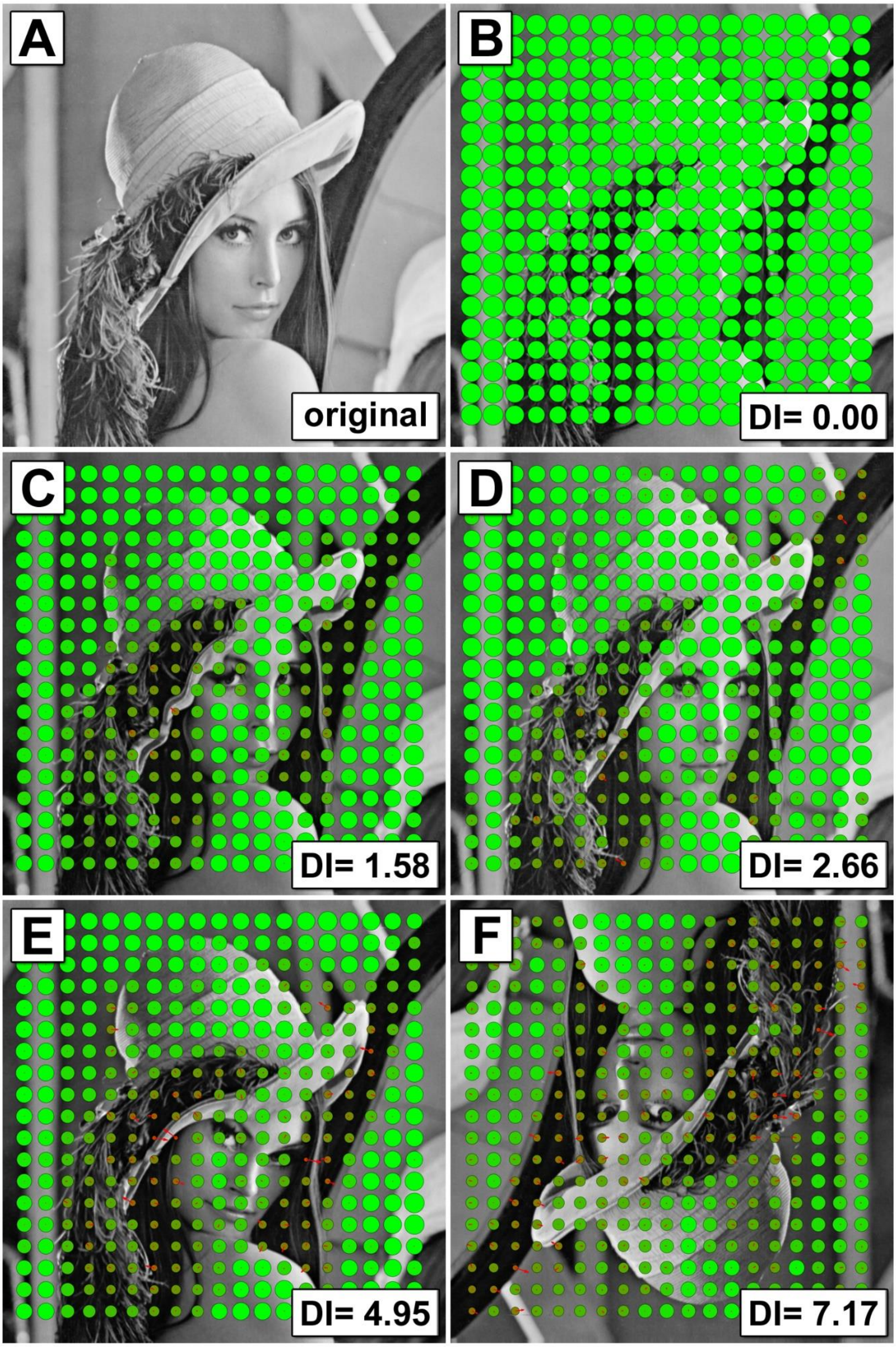

The figure shows the calculation of the Displacement Index (DI) between an example image and a modified version of the same image to validate the DI. (A) Shows the original image, which was scaled to 1024x1024 px and transformed to 8-bit. (B-F) Results of the DI calculations with modified versions of the original image. (B) identity transformation. (C) polar wave field. (D) linear stretching along the y-axis. (E) swirl transformation. (F) rotation of 180°. DI increases with the magnitude of mismatch between the example images.
